# Associations between recollected rates of Category D fruit machine use as a child and adult gambling in a young adult sample

**DOI:** 10.1038/s41598-023-48794-y

**Published:** 2023-12-12

**Authors:** Philip Newall

**Affiliations:** School of Psychological Science, University of Bristol, 12a Priory Road, Bristol, BS8 1TU UK

**Keywords:** Psychology, Human behaviour

## Abstract

There have been recent restrictions to the range of gambling products made available to UK children, with the industry association Bacta introducing a minimum age of 18 for Category D fruit machines, which can otherwise be legally used by children. Any potential government action on Category D fruit machines may want to consider limitations in the current evidence base. The present study addressed this issue, by conceptually replicating previous results associating recollected usage of Category D fruit machine usage as a child and adult gambling. Compared to previous studies, the present study used a younger adult sample, and also tested for potential moderation by subjective confidence. Three out of four main tested associations were significant and in the hypothesised direction, and a lack of significance on interaction terms yielded no evidence of potential moderation by subjective confidence. The present study therefore added to the limited evidence base on potential risks of legal Category D fruit machine usage as a child.

## Introduction

Gambling as a child (aged under 18 years) is a risk factor for experiencing gambling-related harm as an adult^1,2^, and this is one reason why child gambling is an active area of research in the gambling studies field^3–6^. A majority of child gambling research focuses on children’s illegal use of age-restricted gambling products. But children can also gamble legally in a number of ways (e.g., private bets among friends), and legal child gambling might be considered a relatively underexplored topic. The UK has seen recent changes in the availability of gambling products to children, with the legal age of use for National Lottery products being increased from 16 to 18 in 2021^7^. “Category D fruit machines” are another relevant product, which are a class of British traditional electronic gaming machine that has been made legally-available to people of any age^8–10^. Category D fruit machines involve the wagering of money and can pay-out up to £5 in winnings^11^. In 2021 a minimum age of 18 was introduced for the usage of Category D fruit machines with cash pay-outs, not by government (as was the case for National Lottery products), but by the industry trade association Bacta^12^.

It has been noted in other domains that industry self-regulation can be less effective than government regulation^13–16^. For example, an industry body may not have all providers of a given product as its members, or may sanction any providers who fail to meet self-regulatory goals less harshly than government may do. Therefore, as the UK government is currently assessing its approach toward gambling regulation^17,18^, Category D fruit machines are an issue that they may want to consider further. However, any potential government action should also consider the robustness of any underlying evidence base.

Category D fruit machines have been until recently a relatively underexplored area of the UK gambling scene^9^. Two studies have linked higher rates of recollected usage of Category D fruit machine usage as a child with rates of problem gambling severity among adult gamblers^19,20^. Another study replicated this finding and extended it to also find positive associations between rates of recollected usage of Category D fruit machine usage as a child and the probability of being an adult gambler^21^. However, these findings could be due to the retrospective nature of the judgments of childhood Category D fruit machine usage. First, the mean age of these samples were all 28 years or older, and these participants may have struggled to remember events from 10 + years ago. Second, the findings may in part be due to variations in the ability to remember these childhood events across the sample. Adult gamblers, especially frequent adult gamblers, may simply better remember their usage of Category D fruit machines as a child than non-gamblers, perhaps due to the similarity between these events and their adult identities.

Given these valid concerns about the evidence base for associations between childhood use of Category D fruit machines and adult gambling, I aimed to conceptually-replicate previous findings with some design improvements taken from previous research on other gambling products that are legally-available to children. First, the measured associations of H1–H4 below were done in a young adult sample aged 19–23^22^. Second, participants provided a measure of subjective confidence of their recollected childhood use of Category D fruit machines^23^, and interaction models were run to see if differential rates of subjective confidence across the sample could explain any observed positive associations (H5).

The following hypotheses were therefore preregistered:

### H1.

 That any level of recollected engagement with Category D fruit machines, versus not recollecting using them, will be associated with being an adult gambler.

### H2.

 That for adults recollecting using Category D fruit machines, higher frequencies of recalled machine use will be associated with being an adult gambler.

### H3.

 That any level of recollected engagement with Category D fruit machines, versus not recollecting using them, will be associated with higher Problem Gambling Severity Index (PGSI) scores among adult gamblers.

### H4.

 For adult gamblers who recollect using Category D fruit machines, that higher frequencies of use will be associated with higher PGSI scores.

### H5.

 That in models similar to those in H1–H4, but adding a main effect and an interaction for confidence, that the resulting interaction effects will be nonsignificant.

## Method

Data, materials, and the preregistration are available from: https://osf.io/ams2c/. The study received ethical approval from the University of Bristol’s School of Psychological Science Research Ethics Committee (#14,073), and all participants provided informed consent. The methods were carried out in accordance with relevant guidelines and regulations.

An initial 998 responses were collected via Prolific.co, of which 37 (3.7%) self-reported as providing careless responses^24^, so the final sample size used was 961 participants. This initial sample size was selected heuristically based on the availability of research funds. In order to reduce the overall level of recollection biases, only participants aged 19–23 were recruited for the study (*M* = 21.5, *SD* = 1.3). Overall, 487 participants (50.7%) were female (one participant preferred not to say). Demographic information was collected automatically via Prolific.co. Participants were paid £0.40 each, and took an average of 93.3 s to complete the survey (£15.43/hour pro-rata).

The survey involved two blocks presented in random order. In one block, participants were asked if they had gambled in the past 12 months, and participants who responded ‘yes’ then proceeded to complete the PGSI^25^. This approach, of only giving the PGSI to participants reporting gambling recently, is the standard approach used in gambling prevalence surveys^26^. Overall, 606 participants (63.1%) reported gambling in the past 12 months, of whom 234 (38.6% of gamblers) were categorized as no-risk, 183 (30.2%) as low-risk, 131 (21.6%) as moderate-risk, and 58 (9.6%) were in the highest-risk category.

In the second block, participants were provided with a description of Category D fruit machines taken from a previous study^21^, and a relevant image. Participants were asked, “How often do you recall using category D fruit machines while being under the age of 18?”, and responded on a five-point scale of: never, seldom, occasionally, frequently, very frequently^22^. Immediately after, participants provided their level of subjective confidence (“How confident are you in your recollection of this?”) on a scale from 0 (“not at all confident”) to 100 (“very confident”), as first done by another recent study^23^.

H1 and H2 were analyzed via logistic regression, due to the binary nature of the outcome variable (adult gambling, yes/no). H3 and H4 were analyzed via negative binomial regression, as this model can better account for the skewed distribution of PGSI scores than linear regression can^27^. For H5, models were run for all of the significant associations found across H1-H4, by adding main effects for subjective confidence and interactions between subjective confidence and the independent variable for Category D fruit machine use, and interpreted by the significance of the interaction term. A *p*-value of 0.05 was preregistered.

## Results

Descriptive results on frequency of recollected use are shown in Table 1. Overall, 48.7% of the sample recollected using Category D fruit machines at least once as a child; with rates of use being descriptively higher among gamblers (54.0%) than non-gamblers (39.7%). Subjective confidence ratings were also high on average (*M* = 83.4, *SD* = 22.6).

**Table 1:** Descriptive results on frequency of recollected use.

Response	Overall (N = 961) (%)	Gamblers (n = 606) (%)	Non-gamblers (n = 355) (%)
Never	51.3	46.0	60.3
Seldom	29.0	30.5	26.5
Occasionally	15.9	18.5	11.6
Frequently	3.1	4.3	1.1
Very frequently	0.6	0.7	0.6

Results of analyses for H1-H4 are shown in Table 2. As can be seen, support was found for H1 (*Z* = 4.25, *p* < 0.001), H2 (*Z* = 2.16, *p* = 0.031), and H4, (*Z* = 3.68, *p* < 0.001) but not H3 (*Z* = 1.40, *p* = 0.161). Overall, this suggested that merely recollecting using Category D fruit machines was not associated with higher PGSI scores among gamblers (H3). However, usage of Category D fruit machines was associated with being an adult gambler (H1 and H2), and higher rates of recollected Category D fruit machine use were associated with higher PGSI scores among gamblers (H4).

**Table 2:** Main effect models showing associations between engagement and frequency of engagement with adult gambling outcomes.

Effect	Statistic	DV = Adult gambler (Ref = no) Logistic regression	DV = PGSI score (Continuous) Negative binomial regression
Main effect engagement (ref = no)	Coeff (95% CI)	1.78 (1.36; 2.32)	1.19 (0.93; 1.50)
	Z, *p*	4.25, < .001	1.40, .161
	n	961	606
Main effect frequency	Coeff (95% CI)	1.42 (1.03; 1.95)	1.45 (1.19; 1.76)
	Z, *p*	2.16, .031	3.68, < .001
	n	468	327

Logistic regression output are odds ratios, negative binomial regression output are incidence rate ratios.

Output in the left column refer to H1 (top) and H2 (bottom), while output in the right column refer to H3 (top) and H4 (bottom).

Results of analyses for H5 are shown in Table 3, where models add main effects for subjective confidence and interactions between subjective confidence and the main effects for Category D fruit machines used before. An interaction model was not run for H3, due to the lack of statistical significance seen earlier. None of the interaction effects were statistically significant (*p*’s ≥ 0.121), and the estimated coefficients on the interactions were all extremely close to one. This means that H5 was supported; the significant associations found for H1, H2, and H4 did not appear driven by differential levels of subjective confidence.

**Table 3:** Factorial models showing associations between engagement and frequency of engagement with adult gambling outcomes.

Effect	Statistic	DV = Adult gambler (Ref = no) Logistic regression	DV = PGSI score (Continuous) Negative binomial regression
Main effect engagement (ref = no)	Coeff (95% CI)	1.95 (1.46; 2.60) 4.49, < .001	Not run due to insignificance of H3
	Z, *p*		
Main effect confidence	Coeff (95% CI)	1.00 (0.99; 1.01) − 0.07, .945	
	Z, *p*		
Interaction engagement * confidence	Coeff (95% CI)	1.01 (1.00; 1.02) 1.55, .121 961	
	Z, *p*		
	n		
Main effect frequency	Coeff (95% CI)	1.37 (0.98, 1.91) 1.86, .063	1.47 (1.20, 1.80) 3.74, < .001
	Z, *p*		
Main effect confidence	Coeff (95% CI)	1.01 (1.00, 1.02) 2.22, .026	1.00 (0.99, 1.00) − 0.69, .487
	Z, *p*		
Interaction frequency * confidence	Coeff (95% CI)	1.01 (.99, 1.02) 0.86, .390 468	1.00 (.99, 1.01) 0.08, .939 327
	Z, *p*		
	n		

Logistic regression output are odds ratios, negative binomial regression output are incidence rate ratios. Confidence and frequency measures were mean-centered.

Output in the left column refer to H1 (top) and H2 (bottom). A model was not run for H3 (right column, top) due to the insignificance of the main effect. H4 is shown in right column, bottom.

## Discussion

Different approaches have recently been taken with the UK’s range of gambling products that are legally available to children. The legal age of National Lottery products was recently increased to 18 by government regulation, while a minimum age of 18 to use Category D fruit machines was implemented via industry self-regulation^12^. Industry self-regulation can be less effective than government regulation^13–16^, but any potential government action should also consider the robustness of any underlying evidence base. The present results largely supported previous research showing associations between rates of recollected Category D fruit machine usage as a child and adult gambling^19–21^, with three out of four associations significant and in the hypothesized direction. Furthermore, rates of subjective confidence were on average high (*M* = 83.4 out of 100), and zero out of three interaction models were significant, adding further support to these observed associations.

These findings are still subject to various limitations. The measure of subjective confidence only tapped into potential conscious memory distortions, and cannot rule out potential biases that participants were unaware of. Participants were recruited from a crowdsourcing platform, and so this sample was non-representative^28^. The findings are also correlational, meaning that the present results do not show causal effects between Category D fruit machine use and adult gambling. The present study only measured one gambling product, and other products such as coin pusher and crane grab machines are used legally by a high proportion of UK children^29^, which means that the issue of legal availability of gambling products to children is broader than just Category D fruit machines.

In conclusion, the present research largely conceptually-replicated and extended previous findings showing that legal usage of Category D fruit machines as a child is a risk factor for experiencing gambling-related harm as an adult. As a result, UK policymakers may want to consider whether recent industry self-regulation should be reinforced via additional government regulation, where a legal age of 18 for Category D fruit machines would bring them into line with the legal age of use of other gambling products.

## Data Availability

The data are available from https://osf.io/ams2c/.
